# An analysis of the implementation of PEPFAR's anti-prostitution pledge and its implications for successful HIV prevention among organizations working with sex workers

**DOI:** 10.7448/IAS.16.1.17354

**Published:** 2013-03-28

**Authors:** Melissa Hope Ditmore, Dan Allman

**Affiliations:** 1 Independent consultant, New York, NY, USA; 2 HIV Social, Behavioural and Epidemiological Studies Unit; 3 the CIHR Social Research Centre in HIV Prevention (SRC), Dalla Lana School of Public Health, University of Toronto, ON, Canada

**Keywords:** PEPFAR, AIDS, HIV, sex work, prostitution, trafficking, funding, policy

## Abstract

**Introduction:**

Since 2003, US government funding to address the HIV and AIDS pandemic has been subject to an anti-prostitution clause. Simultaneously, the efficacy of some HIV prevention efforts for sex work in areas receiving US government funding has diminished. This article seeks to explain why.

**Methods:**

This analysis utilizes a case story approach to build a narrative of defining features of organizations in receipt of funding from the President's Emergency Plan for AIDS Relief (PEPFAR) and other US funding sources. For this analysis, multiple cases were compiled within a single narrative. This helps show restrictions imposed by the anti-prostitution clause, any lack of clarity of guidelines for implementation and ways some agencies, decision-making personnel, and staff on the ground contend with these restrictions.

**Results:**

Responses to PEPFAR's anti-prostitution clause vary widely and have varied over time. Organizational responses have included ending services for sex workers, gradual phase-out of services, cessation of seeking US government HIV funds and increasing isolation of sex workers. Guidance issued in 2010 did not clarify what was permitted. Implementation and enforcement has been dependent in part on the interpretations of this policy by individuals, including US government representatives and organizational staff.

**Conclusions:**

Different interpretations of the anti-prostitution clause have led to variations in programming, affecting the effectiveness of work with sex workers. The case story approach proved ideal for working with information like this that is highly sensitive and vulnerable to breach of anonymity because the method limits the potential to betray confidences and sources, and limits the potential to jeopardize funding and thereby jeopardize programming. This method enabled us to use specific examples without jeopardizing the organizations and individuals involved while demonstrating unintended consequences of PEPFAR's anti-prostitution pledge in its provision of services to sex workers and clients.

## Introduction

Funding from the President's Emergency Plan for AIDS Relief (PEPFAR) has enabled access to treatment for more than 3.9 million people living with HIV and AIDS in places where they would not otherwise have received anti-retroviral medicines (ARVs). In the fiscal year, 2011, PEPFAR supported HIV testing and counselling for more than 40 million people, including 9.8 million pregnant women. However, not all PEPFAR funding is for treatment and testing. A high number of HIV prevention programmes around the world receive PEPFAR funding also [1].

Since 2003, US government funding to address the HIV and AIDS pandemic has been subject to an anti-prostitution clause – colloquially known as “pledge” – that requires aid recipients to adopt an organizational policy opposing prostitution. In essence all who agreed to receive PEPFAR funds were required to state in writing that any and all activities supported by PEPFAR would not encourage or condone prostitution. From its inception, the pledge was accompanied by a second restriction forbidding the “promotion of prostitution” by grant recipients. This second restriction has been presented by the US administration as a mechanism to prevent the promotion of prostitution and human trafficking through its donor monies. This article seeks to demonstrate that despite this intent, the pledge has blossomed into something altogether different when implemented in the field [2]. This article first documents the history of the pledge and then applies a case story method to illustrate the unintended yet adverse effects of the implementation of the pledge.

### Origin and history of the anti-prostitution policy

The anti-prostitution clause as it presently exists within US policy has a convoluted and nuanced history (see Table 1) [3–24]. In December 2002 Colin Powell, the United States Secretary of State under President George W. Bush issued a directive that organizations in receipt of US government funding should remove references to condoms from their websites, and also that “organizations advocating prostitution as an employment choice or which advocate or support the legalization of prostitution are not appropriate partners for USAID anti-trafficking grants and contracts, or sub-grants and sub-contracts” [25]. This foreshadowed the inclusion of a clause indicating that “no funds … may be used to provide assistance to any group or organization that does not have a policy explicitly opposing prostitution and sex trafficking” in the United States Leadership against HIV/AIDS, Tuberculosis, and Malaria Act [3] (known as the Global AIDS Act), passing, in May 2003. The text of the act also states that “no funds made available to carry out this Act, or any amendment made by this Act, may be used to promote or advocate the legalization or practice of prostitution or sex trafficking” [3]. A similar restriction was included in the Trafficking Victims Protection Reauthorization Act of 2003, passed prior to the Global AIDS Act, stating that, “No funds made available to carry out this division, or any amendment made by this division, may be used to promote, support, or advocate the legalization or practice of prostitution” [26]. Requirements of anti-prostitution policies have become standard in US funding agreements, and two lawsuits have been filed against these restrictions as applied to HIV prevention programmes [27]. Nonetheless, the inclusion of such restrictions has had a profound impact. For example, in July 2004, a statement issued by the Centers for Disease Control and Prevention of the US Department of Health and Human Services aimed at the expansion of HIV and AIDS activities for vulnerable populations in Côte d'Ivoire indicated that any foreign recipient of aid monies needed to have in place a policy explicitly opposing prostitution and sex trafficking [28].

**Table 1:** PEPFAR anti-prostitution pledge timeline

Date	Event	Notes
May 2003	Global AIDS Act signed [3].	
January 2004	The Global Fund for AIDS, Tuberculosis and Malaria, the World Health Organization, the International AIDS Vaccine Advocacy Coalition, and all UN agencies are exempt from the pledge [4].	These exemptions are made clearer in language released by the CDC in May 2005, stipulating that these organisations are not subject but that sub-grantees are subject to the pledge.
2004	US Office of Legal Counsel opinion about enforcing the pledge upon US-based organisations working abroad written but not publicly released in its entirety. The Brennan Center filed a FOIA request for this document [5].	
March 2005	Statement appears: “U.S. law … prohibits such funds from being used to implement any program that targets victims of severe forms of trafficking in persons involving sex trafficking by an organization that has not stated in either a grant application, a grant agreement, or both, that it does not promote, support, or advocate the legalization or practice of prostitution. It is the responsibility of the primary grantee to ensure these criteria are met by its sub-grantees” [6, see also 7].	
June 2005	Department of Justice reverses earlier First Amendment based ruling.	
June 2005	USAID issued a directive that only organisations with an explicit policy against prostitution and sex trafficking should be funding recipients. Guidelines for funding include the right of USG representatives to investigate activities to enforce the pledge. Guidelines further state that funding recipients may have partners including subcontractors that do not have policies provided there is “sufficient” separation, reminiscent of the separation required under the Global Gag Rule addressing abortion, which was not defined but instead was addressed on a case-by-case basis [8, 9].	
August 2005	DKT files suit against USAID to challenge the anti-prostitution policy requirement, saying. “DKT has no policy on prostitution and does not wish to adopt one” [10].	
September 2005	AOSI/Pathfinder lawsuit filed contesting the pledge.	
January 2006	BBC rejects US funding with the pledge [11].	“But six months into the contract, the US government terminated the project after tightening up on a requirement that organisations receiving US funds had to sign a pledge “explicitly opposing prostitution.” The BBC project would not have provided direct services to Tanzanian prostitutes, but some programmes might have dealt non-judgmentally with their role in the epidemic. A signature on the anti-prostitution pledge would have entitled US government officials to vet all the trust's projects worldwide for compliance with Washington's “morality” doctrine. The BBC's Tanzanian project would also have had to join the US campaign to promote sexual abstinence by stressing the failure rates of condoms.”
April 2006	US Government Accounting Office (GAO) releases report “Spending Requirement Presents Challenges for Allocating Prevention Funding under the President's Emergency Plan for AIDS Relief” [12].	Report criticizes promotion of ideological (abstinence and monogamy) rather than evidence-based, proven-effective programming. Report also cites lack of clarity about what could be done to make condoms accessible and the confusion
		created by spending earmarks that diverted money to abstinence programming at the expense of other programmes.
May 2006	District court rules that the anti-prostitution pledge violates First Amendment rights in DKT v. USAID.	
May 2006	District court rules that government must stop requiring plaintiff organisations to comply with anti-prostitution pledge. This applies only to organisations that signed on to the AOSI/Pathfinder lawsuit. These organisations number over 200.	
February 2007	US Circuit Court reverses DKT v. USAID ruling and upholds anti-prostitution pledge.	
March 2007	Institute of Medicine releases report, “PEPFAR Implementation: Progress and Promise,” calling for greater emphasis on prevention of HIV infection generally, improved data on prevalence and at-risk populations [13].	
January 2008	Reauthorization of US anti-trafficking legislation [14].	
February 2008	Congress discusses the reauthorization of PEPFAR, and a coalition of NGOS successfully advocated for the removal of the anti-prostitution pledge in the original drafting of the legislation. Representative Tom Lantos (D-CA) championed an end to the abstinence earmark and the anti-prostitution pledge, saying “It is inconsistent with this goal to place ideologically driven restrictions on the implementation of efforts to prevent spreading the virus.” Lantos died after a long illness. However, the anti-prostitution pledge was reinstated during closed-door sessions between right-wing Christian conservative Representative Chris Smith (R-NJ) and some NGOs [15, see also 16].	
July 2008	The Lantos-Hyde Act reauthorized PEPFAR [17] with a significant increase in funding but retaining anti-prostitution pledge signed by then-US President George Bush. The abstinence earmark was removed but requires reporting justification of spending less than half the funds on abstinence and be faithful programming (the A and B of ABC).	
August 2008	Court grants injunction against anti-prostitution pledge requirement.	
December 2008	Guidelines for anti-prostitution pledge require grantees to “certify” their “objective integrity and independence from any organization that engages in activities inconsistent with a policy opposing prostitution and sex trafficking” [18].	
June 2009	UN Secretary General Ban-Ki Moon states “In countries without laws to protect sex workers, drug users, and MSM, only a fraction of the population has access to prevention. Conversely, in countries with legal protection and the protection of human rights for these people, many more have access to services. As a result, there are fewer infections, less demand for anti-retroviral treatment, and fewer deaths. Not only is it unethical not to protect these groups; it makes no sense from a health perspective. It hurts all of us.” in his statement to the International AIDS Conference [19].	
May 2009	Court rules in AOSI/Pathfinder suit that the pledge violates the First Amendment rights of the plaintiffs. This applies only to the organisations signed on to the suit, including members of the Global Health Council.	
January 2009	President Barack Obama takes office and begins appointing members of the administration.	
July 2009	Eric Goosby, US Global AIDS Coordinator, states that PEPFAR will seek to use human rights based approaches to sex workers, as well as MSM and drug users, during his address to an International AIDS Society meeting [20].	
November 2009	Eric Goosby, US Global AIDS Coordinator, says “My role is to be supportive and helpful to the patients who need services. It is not to tell a country how to put forward legislation. But I will engage them in conversation around my concern and knowledge of what this is going to do to that population.” in response to uproar over PEPFAR support to Uganda which implements an actively homophobic agenda. Note the lack of clarification of the word population as used by Goosby [21].	
April 2010	Department of Health and Human Services releases new guidance on implementation of anti-prostitution pledge. The new regulation requires recipients to “agree” that “they are opposed to the practices of prostitution and sex trafficking because of the psychological and physical risks they pose for women, men and children” [2: 45 C.F.R. § 89.1(b), 22].	
April 2010	The new guidance also makes some adjustments to what determines adequate separation from a sub-grantee doing work that may be constrained by the pledge. Legal separation is still a factor, but physical separation is required “to the extent practicable in the circumstances.” Itemized separation is no longer stated but consideration of these and other factors remain at the discretion of HHS [2: 45 C.F.R. § 89.3(b), 22].	
July 2010	During the International AIDS Conference in Vienna, Austria, Eric Goosby, US Global AIDS Coordinator, states that sex workers would be “embraced” at all US funded HIV and AIDS services, and that if discrimination against sex workers were to occur at any such programme, the US government would be “on that like a laser” [23].	
January 2013	The Supreme Court of the United States announces that it will hear the case challenging the pledge [24].	

**Table 2:** Case story

	Organization: Agency X	Organization: HQ	Organization: HOBO
Status	Large international NGO based in the US with in-country office and programmes	Largest non- international NGO In the nation	Local, small NGO with small staff, and locally driven programmes
Funding Sources	USAID, multilateral donors, other national donors	Sub-grantee of Agency X, sometimes a direct recipient from USAID, including PEPFAR	Partner with NGOs across the country, sometimes a sub-grantee from USAID and funding from private foundations
Description of SW project at the time the pledge was instituted	Runs a programme for sex workers Some sex workers are in low paid positions with intention to train and promote them	Runs extensive programme with drop-in centres for sex workers, including health services Conducts social marketing of condoms and personal lubricant Supports sex workers’ anti-violence campaign by offering them space to meet	Has a small, well-respected sex work project, that is very much community-led aside from medical services HIV programming, meetings of sex workers, support of self-organizing of sex workers Some healthcare is offered on-site. Referrals are made to other existing services
Introduction of pledge	Stops publicizing sex work project Cancels planned scale-up of successful sex work project	Meets with USAID country officer, who explains that what they understand is that drop-in centres are not allowed Drop-in centres for sex workers close Other HIV programming continues to provide services to sex workers, including social marketing of condoms and lubricant Staff would like to stop working with sex workers, because they are stigmatized for working with sex workers but management does not want to end programming	Programming does not change. Information sharing continues
Response to 2006 investigation of sex work projects at a US based NGO	Divestment from SW programming becomes a priority Seeks to spin off SW project, but there are no organizational takers because of the climate after the pledge	Stops seeking HIV funding Ends social marketing of condoms and lubricant Personal lubricant becomes virtually unavailable	Discussion of what this means for SW project. No changes to programming services Information about project shared
Response to 2006 investigation of sex work projects at a US based NGO	Community members employed by project lack skills necessary to run project. Debate about whether capacity building among sex workers to run the project themselves would be considered “promoting prostitution” prevents training in skills necessary to run an organization Funding is secured from another agency for the SW project SW project is spun off, with one staff person from Agency X moving to it	End of support for sex workers’ anti-violence campaigns. Support was in the form of meeting space	
2008	Spin-off SW project eventually closes due in part to the lack of organizational skills among sex worker staff without capacity building and training		Information about project shared only with local partners
2009		Seeks HIV funding again. Sex work programming is not included	
2010 IAC			Not publicizing work with sex workers, despite strong programming, in deference to pledge and lack of guidance about what could be construed as “promoting prostitution”

Funding was authorized in 2004 with a call for proposals, which stated “No funds made available … may be used to provide assistance to any group or organization that does not have a policy explicitly opposing prostitution and sex trafficking” [29]. This was reiterated in 2005, when USAID issued a directive that only organizations with an explicit policy against prostitution and sex trafficking should be funding recipients and also when the pledge was first applied to US-based organizations. At this time, the government of Brazil declined US$ 40 million of these conditional funds because they would inhibit HIV prevention programming [30]. Some organizations also have declined such conditional funding. For example, the BBC declined this conditional monetary support [31] for an HIV-oriented programme. Additionally, the US government made a concerted effort to fund faith-based organizations, which were typically new to HIV and AIDS programming and generally less familiar with evidence-based HIV programming [32]. While all grant recipients developed organizational policies as required by the pledge, organizations that were less familiar with implementing evidence-based programming may have been less likely to consider the implications of the enactment of the pledge.

In 2008, efforts to remove the pledge were defeated [33] and US Congress re-authorized PEPFAR with a significant increase in funding, but no change in requirements. Grantees were still requested to “certify” their “objective integrity and independence from any organization that engages in activities inconsistent with a policy opposing prostitution and sex trafficking” [34]. In 2010, under the Obama administration, the US Department of Health and Human Services released new guidance on the implementation of the anti-prostitution pledge, requiring grantees to “agree,” in a phrasing of the anti-prostitution pledge embedded within PEPFAR contracts, that the recipient is “opposed to prostitution and sex trafficking because of the psychological and physical risks they pose for women, men and children” [11].

This was arguably less difficult to meet than some of the previous requirements because the policy had been written into the contract in a form that was more like a philosophy rather than a policy developed and implemented by the organization. Nevertheless, organizations are known to continue to decline funding that includes such a clause. One example is SANGRAM, an organization for sex workers in Sangli, India, known for its community empowerment model that addresses needs identified by the sex workers served by the organization [2]. Another example is found in a short item in *The New York Times Magazine* about an organization of sex workers in Cambodia that turned down a substantial grant, asking: “Do they think we're worse than dogs?” [35]. For those for whom declining funding is not an option, the outcome is that organizations struggle to provide a dignified and effective service to a population that they are encouraged to “oppose” and as a result may find they are unable to deliver services without stigmatizing their intended beneficiaries.

Despite the origin of the anti-prostitution pledge in a broader policy context, we focus specifically on PEPFAR because this is the pool of money that most directly affects the greatest number of sex workers, as members of what USAID terms a “most-at-risk population” (MARP) [17]. The United States is the largest national donor for HIV-related funding, with over US$ 4 billion approved funding in 2010 [25]. US funding for other programmes affecting sex workers, such as anti-trafficking money, is dwarfed by the funds for PEPFAR (for example, $16 million for anti-trafficking programming abroad in fiscal year 2011 [15]). However, the anti-prostitution clause is not applied exclusively to PEPFAR funding only, but to all the organizations’ funding, and therefore affects education and other programming. In this way, these funding restrictions affect many more programmes in addition to HIV-related programming, including programmes promoting access to clean water, sanitation, life-saving medicines and medical care. These requirements and the subtle changes to their contract wording have far-reaching implications because they affect the distribution of many millions of aid dollars.

The pledge has received strong support from activists and politicians in the United States who take a philosophical or religious stance against prostitution. For example, the chair of a women's studies department at a state university gave a speech attacking individuals and organizations working with sex workers for addressing the concerns of sex workers including HIV and AIDS rather than working to eliminate prostitution [36]. Public support for the anti-prostitution clause has led to investigations of projects in receipt of PEPAR funds by conservative Christian politicians; this is one example: after the aforementioned speech was given, a project in Cambodia was closed [37] seemingly in response to an investigation directly linked to the speech. Once the Cambodia project was investigated and eventually closed, an organization of local sex workers that had been part of a network of HIV and AIDS projects in Cambodia was suddenly isolated from their contacts within other programmes and excluded from this network of HIV and AIDS projects [38].

### What do these restrictions mean in programming?

Specific activities prohibited by this restriction have never been defined; rather, guidance has been vague. This vagueness has led to arbitrary and unsystematic interpretations of the pledge, contributing to self-censorship by grant recipients. This article offers information from the field about how funding restrictions have been implemented, particularly in HIV programming. In this article, we offer examples from actual programmes which have been anonymized using a case story methodology in order to argue that the policy has compromised the efficacy of US-funded HIV prevention efforts, particularly with regard to most-at-risk populations including sex workers and transgender people.

## Methods

This analysis utilizes a case story approach [39] to build a narrative of defining features of organizations in receipt of PEPFAR funding. The case story approach is ideal for working with data that are highly sensitive and vulnerable to breach of anonymity because it limits the potential to betray confidences and sources [40], and in this context, jeopardize funding and programmes. The approach also allows construction of a narrative that more fully represents the impact of the funding restrictions within PEPFAR contracts than a case study of a single organization could. For this analysis, therefore, multiple cases are compiled within a single narrative in order to allow identification of restrictions imposed by the anti-prostitution clause, lack of clarity of guidelines for implementation and ways some agencies contend with these restrictions.

Whereas the use of a case study methodology is common within medical research and other disciplines [41–43], the application of a case story methodology is much more novel. A case story differs from the more frequently-used case study in that typically a case study uses one sole case as an illustrative and defining example. In contrast, a case story compiles numerous examples into one narrative. It does so by selecting various narrative elements from multiple cases and compiles them into a single case.

Our information has been collected since 2003 and comes from published accounts and directly from sex workers, NGO staff and USAID representatives, working on five continents. These reports come from over 25 organizations and projects in 14 nations in Africa, the Asia-Pacific region, the Americas and Europe. As the authors’ interest in PEPFAR and funding restrictions became known through publications (e.g. [25, 44–46]), some people came forward without prompting to share information with us.

Case stories use multiple accounts in order to triangulate events and to identify generative themes or recurring tensions [47]. We present these scenes in this narrative because they are representative of the experiences of organizations and people. Each event presented actually happened in at least one location, and there was significant overlap and multiple occurrences of many of the events presented. However, to the best of our knowledge not all events in this case story have occurred in any single location. This case story uses three organizations, each a composite, to illustrate different strategies and responses to US funding policy. This case story incorporates examples from the people and projects that have shared information with the authors to illustrate the ways these restrictions have affected sex workers.

## Results: the case story

### Background

In the context of our case story, set as it is in the fictional country of West Lannadesh, NGOs do not promote prostitution or trafficking in persons. Rather, NGOs advocate for the health and wellbeing of their constituents and work to prevent social and structural harms (see Table 2). Within this context many if not most stakeholders are unsure of what the PEPFAR policy means. This is in part because the guidance that has accompanied the PEPFAR policy has been unclear about what exactly “promotion” means. This has resulted in some NGOs being falsely accused of encouraging sex work and trafficking, which has been exacerbated by widespread conflation of human trafficking with sex work. In the case story – reflective as it is of actual events – many organizations have restricted their activities in response to the anti-prostitution policy requirement. Indeed, this has been the experience in the fictional country of West Lannadesh.

### Setting

West Lannadesh is a poor country with limited manufacturing and a growing population. Neighbouring countries include a nation that has signed the US anti-prostitution pledge and a middle-income nation that has rejected a substantial but not overwhelming amount of US funding because of the pledge.

### Description of sex work environment

Sex work is carried out in a wide variety of venues including streets, bars, hotels and brothels. The overwhelming majority of clients are locals. Some bars cater to Western aid workers and a small number of highly visible tourists. Condoms are accessible through social marketing programmes, some of which employ sex workers. The condom sales support some of the local sex work organization's staff.

### Description of HIV epidemics

HIV prevalence is around 1%, with a concentrated epidemic of HIV among sex workers and people who use drugs, around 15%. It is suspected that hepatitis C virus (HCV) co-infection is high but testing is infrequent and treatment for HCV is not available. Incidence is highest among people under 35 years of age.

### Description of access to prevention, treatment, care and support

Only people who can afford to pay for HIV-treatment or other medical care receive any kind of treatment outside of NGO settings. The very wealthy leave the country for medical care. Most people receive no treatment and visit pharmacies for medical needs only when they absolutely must. However, PEPFAR has made it possible for approximately a quarter of the people who need HIV medications to receive them, when their CD4 counts dip below 200.

Agency X is a large international NGO based in the US. Agency X has many programmes, most of which focus on development. Their programmes with sex workers earn high praise for their innovative, community-based approaches and success reducing the incidence of HIV among sex workers. Efforts to scale up these programmes have stalled since the imposition of PEPFAR's anti-prostitution policy. In fact, all plans for scaling up evidence-based programming with sex workers have been abandoned. Projects with sex workers remain small “boutique” projects that are sound but not publicized in reports from Agency X. Agency X seeks to divest itself from sex work programming in a responsible way and is therefore trying to ensure that these small, evidence-based programmes carry on without being associated with Agency X. This divestment is a priority for Agency X because of the investigation of a similar organization for work with sex workers. Congressional staff was physically present in the offices of this other agency, and their questions about particular projects demanded so much time that the headquarters office was fully occupied in submitting to their requests. This investigation led to an inability to fulfil contracts during this three-month period. Agency X is one of a number of organizations attempting to extricate themselves from working with sex workers. Their decision was directly influenced by this investigation of another organization.

Agency X tries to encourage and train local organizations to take over these programmes with sex workers, including seeking alternative funding for them. Agency X's efforts to divest from its work with sex workers are made difficult because partners hesitate to take on programming that is itself stigmatized and which may prevent their seeking large grants from the US government. Many of the people who benefit from the programme would like to work for it and ensure its continuity, but most beneficiaries are not literate and have had little if any formal schooling. Several years of capacity building would be necessary for the sex workers themselves to run all aspects of the organization, but Agency X and others are concerned that this capacity building would be considered to promote prostitution. In this situation, these programmes are destined to stay small and remain marginalized within an organization, possibly Agency X but possibly not, or be conducted by an organization that is itself marginalized by other NGOs, even or perhaps especially if the sex workers themselves run the project.

Agency X is one of a number of organizations in West Lannadesh, many of which are trying to navigate their way through the anti-prostitution pledge. The largest local organization, HQ, works in partnership with smaller organizations, some of which are subsidiaries. It operates in an extremely violent post-conflict setting with histories of civil war and natural disasters. It has been in existence for 22 years. HQ operates a series of clinics in a large urban area, in partnership with international aid agencies and local and foreign universities. The mandate of HQ is HIV education, prevention, care and support. This includes an array of services that individuals can access including a drop-in centre, bathing and sanitation facilities, and condom distribution. HQ operates under a variety of funding arrangements, one which is a multi-year grant provided by USAID. One condition of this grant is the requirement to sign PEPFAR's anti-prostitution pledge. The Director of HQ is clear that the agency does not support prostitution or sex trafficking. Although the organization does not condone prostitution, it does not condemn the individuals involved in it. The result is the pledge has led to conflict between HQ management and its staff as sex workers have traditionally come to HQ to use the drop-in centre, to bathe and to get condoms. It is not clear to the HQ director or staff how this would be supporting prostitution.

Despite this, some HQ staff are in favour of the pledge. For example, there are doctors who work at the HQ clinics who see the pledge as an opportunity to withhold services from sex workers. These individuals rationalize this to colleagues by suggesting that they never wanted to work with prostitutes and that the pledge will lead to great personal relief, as they will no longer be required to experience the stigma of working with these people. Sex workers are highly stigmatized, so much so that this stigma affects those providers who work with sex workers, including in healthcare settings.

The Director of HQ consulted with the local USAID Country Officer who clarified that drop-in centres for sex workers are definitely not permitted under this restriction. At the same time, the Country Officer confides to the Director that this is one of the few areas that is specific and clear regarding this policy. Rather than police who can and cannot use the HQ drop-in centres, HQ simply closed them. In addition, any clinic attendees who are known or suspected to be involved in sex work are informed that they will no longer be provided services. The result is that sex workers are publicly forced to leave the clinic. This was the only clinic they attended in the city because others, including public clinics, either barred them outright or made them so unwelcome that sex workers did not return. There are no other services dedicated to sex workers. Therefore, HQ's denying sex workers services effectively denies them any healthcare.

With no drop-in centre access, sex workers have no place to get off the street. Further, homeless sex workers have no access to basic bathing and sanitation facilities. In response to the pledge, local sex workers organize a meeting with the staff of an international human rights organization. They report that HQ has informed them that in addition to exclusion from its clinics and the closure of its drop-in centres, HQ will no longer support sex workers organizing against violence by offering a space for sex workers to meet and strategize anti-violence efforts, despite the fact that sex workers and HQ had worked collaboratively together for 12 years. Local sex workers cannot understand these developments, as they are sure that all HQ and local partner staff are against violence. The international human rights organization makes it clear that it is not violence that is the issue but rather the new restriction on HQ and partners imposed by the anti-prostitution pledge and that the restriction makes HQ leadership worry whether supporting this anti-violence effort could be construed as promoting prostitution.

Under increased pressure from sex workers, local communities and human rights organizations, and worried whether programming that continued after the closure of the drop-in centres could be considered promoting prostitution, the board of HQ decides to stop seeking HIV funding, and instead to concentrate on school-based sexual education, which is not affected by the US anti-prostitution pledge, but which does not reach sex workers. Peer education and social marketing of condoms and safe sex supplies for sex workers is halted. Locally, condom sales drop by more than two-thirds. Personal lubricant becomes almost inaccessible because the cost is simply prohibitive outside social marketing schemes.

Realizing that a dangerous situation regarding human rights and public health seems imminent, HOBO, an altogether different outreach organization, that is also in receipt of PEPFAR funds agrees to allow sex workers to meet and to organize at one of their satellite offices, provided that the sex workers do not make the information regarding their meetings public. When asked how HOBO can be sure that they will not be punished under the PEPFAR policy, an employee of HOBO indicates that the NGO is a small part of a much bigger project and that if USAID officials visit, they will not be told about the sex worker group. Internally, and in time, HOBO staff members are praised for the increase in the use of its services and the improvements in its outcome evaluations. Unlike the Director of HQ, the Director of HOBO indicates to its staff that information pertaining to sex workers’ use of services will not be published and will be made available only to its staff.

At the 2010 World AIDS Conference, an informal discussion is held among Directors of outreach organizations similar to HOBO. There, a colleague from a neighbouring country indicates to HOBO's Director that while they were looking forward to seeing the HOBO annual report, in particular, to the outcomes of the new and covert services delivered to sex workers that, not reporting this information was the right thing to do: “I understand. My organization has adopted a similar policy. I only wish we could do more.”

## Discussion

Government and NGO personnel report that the guidance on the implementation of the anti-prostitution pledge has been unclear and enforcement has been unpredictable. As described above, written guidance has not stipulated what exactly is or is not permitted, and therefore, many organizations have interpreted the restrictions on their own, with varying results. While some organizations’ personnel, as at Agency X, advised declining to work with sex workers altogether for fear of losing the important USAID contract, others cautioned that in doing so, sex workers would be discriminated against and denied critical HIV prevention and health services. The consensus reached by one organization, HOBO, was to modify its terminology in order to offer services to sex workers without compromising their US funding, and when appropriate, to implement USAID's non-discrimination clause that prohibits the denial of services to anyone, but also to attempt to suppress and withhold information about their work with sex workers.

In some instances, one of the effects of funding restrictions is that programming has been eliminated. Within some organizations, peer education for sex workers about safer sex techniques have ended. Campaigns against violence against sex workers, who are subject to some of the highest rates of violence among any population have been dropped.

NGO staff have used restrictions to promote their prejudices. This reinforces stigmatization and discrimination. Some NGOs no longer serve some of the least-served and most-at-risk people, sex workers. Those who are homeless, transgender, or otherwise suffer double stigma are most adversely affected. A large number of NGOs have limited their funding and programming and even their discussions in wide-ranging self-censorship to ensure that they remain within this limitation. Reports and publications about successful HIV and AIDS programming with sex workers have been suppressed.

## Conclusions

### Breadth

These are restrictions that apply to many programmes, beyond USAID, as PEPFAR is the largest US foreign aid programme. The restriction's wide scope inhibits the sharing of information in the form of reports, papers, presentations and other media. The lack of information sharing is a direct result of the chilling effect of the restrictions. Furthermore, the lack of information sharing prevents the development, implementation and replication of effective programming for sex workers, far beyond PEPFAR.

### The anti-prostitution pledge works counter to HIV prevention

Best practices for HIV prevention emphasize combating stigma and discrimination [48] and the involvement of target populations in designing effective programs [19, 49]. Sex workers’ descriptions of the adverse effects of this restriction have been repeatedly ignored by multiple US administrations. This is counter to best practices and should be rectified for evidence-based proven-effective programming.

While PEPFAR has made life-saving medicines accessible to many, our research documents that it has also promoted the stigmatization of sex workers and discrimination against sex workers.

New guidance issued in April 2010 does not clarify what is permitted or restricted and so cannot rectify this situation created in part by lack of guidance [22]. The new guidance further promotes stigma and discrimination by discussing unproven effects. PEPFAR's August 2011 HIV prevention guidance states that “there is substantial evidence for the effectiveness of a core set of interventions for populations at high risk of HIV, including [sex workers]”. However, there is no clear guidance for HIV prevention programming with sex workers, although there is such guidance for other populations at higher risk of HIV including men who have sex with men [50] and people who inject drugs [51].

PEPFAR's anti-prostitution pledge has had unintended consequences on NGOs, local organizations and primarily, the provision of services to sex workers and clients. Outreach staff indicate that HIV prevention has been less successful since the inclusion of the pledge, and local HIV Incidence rates reflect this, particularly among sex workers and people presumed to be sex workers, including some gay men and transgender people. The funding restrictions have reduced or eliminated access to services. Drop-in centres have closed; in some instances sex workers no longer have access to places to bathe and use a toilet. Sex workers have been denied clinic services. Sex workers have less access to condoms and personal lubricant, critical HIV prevention tools and necessary commodities for safe sex. The current US Global AIDS Coordinator has been quoted as saying that turning away anyone who should receive services would not be tolerated:

What the clause really was focused on was to ensure that PEPFAR did not fund organizations involved in trying to legalize prostitution and traffic women into prostitution. We have changed it so an organization doesn't have to sign [a separate document pledging to oppose sex work and sex trafficking]; we have folded in an agreement that the [beneficiary] organization will not traffic women into prostitution – there is no separate document. PEPFAR has not de-funded any programme on the planet for these reasons. We want to care for every sex worker out there. If a sex worker comes into any of our facilities, that person will be embraced and followed for the duration of their life on anti-retrovirals. If there are examples of anybody being turned away [for being a sex worker], if someone feels that they were excluded from or dropped out of care for those reasons, we would get on that like a laser [23].

This statement implies that the US government wishes to enforce the anti-discrimination clause that prevents anyone, including sex workers, from being denied services. However, this type of clarification does not address or counter the preceding years’ promotion of discrimination against sex workers or organizations working with them, which cannot be undone through popular reportage, particularly considering the phrasing in US contracts through 2011 and lack of clear guidance about what exactly is and is not permitted with US funding. This lack of clarity allows the US administration to continue to enforce the pledge as it sees fit, as the previous administration has and the next administration could. The provision of life-and-death services should not be determined by “political winds” [52]. Clear guidance for HIV programming with sex workers could alleviate this.

Clearly within policy arenas there are mixed perspectives on the impacts of the PEPFAR restrictions with regards to sex work. However, real world prevention care and treatment of HIV and AIDS for sex workers does not occur in policy arenas but rather on the ground, in venues and avenues far removed from policy discourse. Meanwhile, although policymakers reflect on the intent or the outcome of the pledge, on the ground the policy has and may continue to have consequences. As a result of the pledge, in many instances information sharing about successful programming with sex workers has nearly ceased. Sex work programming has become a taboo topic; organizations that receive other funding are likely to be interested in or to seek US government contracts and funds. Others with specific missions have reigned in all activities unrelated or tangentially related to their missions; this has affected many sex work projects the world over. The anti-prostitution pledge has prevented the sharing of information about successful programming and prevented scaling up successful operations.

The US government should reconsider this funding restriction in the light of diminished effectiveness, and instead implement evidence-based interventions in its HIV programming.
